#  Carbon Nanotubes: a Promising Approach for Drug Delivery 

**Published:** 2010

**Authors:** Reza Aboofazeli

The bioavailability and intrinsic toxicity of many potential drugs affect their pharmacological profile and, in turn, their therapeutic efficacy and therefore, preventing them from late-stage pharmaceutical development. Hence, pharmaceutical industries are recently being forced to renew creatively their drug discovery programs and design more and better drug candidates for clinical development. Incorporation of nanotechnology at the early stage of drug development process could be considered as a potential source of new drug design strategies. During the past few decades, remarkable advances in particle engineering and design of nanoscale drug delivery systems have been occurred, such that recently, nanotechnology has been used successfully in the design of particulate systems with uniform particle size, desired surface characteristics and geometrical forms. The great advantage of nanotechnology is its potential capability of manipulating and modification of the arrangement of single atoms and molecules and creating unique structures with unique characteristics, leading to an improvement of membrane penetration, increase in solubility of poorly soluble drugs, increase in stability of biomolecules and their bioavailabilities and consequently an improvement in drug delivery efficiency. 

One of the most recent strategies proposed to incorporate nanotechnology principles is through the application of carbon nanotubes (CNTs), which leads to the modulation of undesired effects and creating new conjugates with promising and improved pharmacological profiles. CNTs have been proposed and actively explored as multipurpose innovative carriers for drug delivery and diagnostic applications. Their intrinsic physicochemical features enable covalent and non-covalent binding of several pharmaceutical entities and allow for rational design of novel candidate nanoscale structures for drug development. CNTs can be functionalized with different functional groups to carry simultaneously several moieties for targeting, imaging and therapy. 

**Figure 1:**
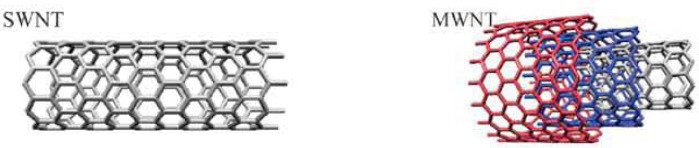
Structures of SWNTs and MWNTs carbon nanotubes

The distinct structural properties of carbon nanoparticles, in particular their high aspect ratio and propensity to functional modification and subsequent use as carrier vectors, as well as their potential biocompatibility and nanofluid nature, make them useful for nanodelivery and controlled drug delivery. CNTs are tubular objects with a relatively well-defined diameter in the range of nanoscale with controllable lengths, classified as single-walled (SWNTs) and multi-walled (MWNTs) structures (Figure 1). CNTs belong to fullerene family of carbon allotropes. They are cylindrical molecules consisting of a hexagonal arrangement of sp^2^-hybridized carbon atoms and are described as hollow cylinders formed by rolling single or multiple layers of graphene sheets into cylinders. SWNTs are composed of a single cylindrical graphene layer capped at both ends in a hemispherical arrangement ofcarbon networks, while MWNTs comprise of a varying number of concentric SWNT layers. MWNTs generally have a larger outer diameter than SWNTs. SWNTs have a better defined diameter, whereas MWNTs are more likely to have structural defects, resulting in a less stable nanostructure. 

**Figure 2:**
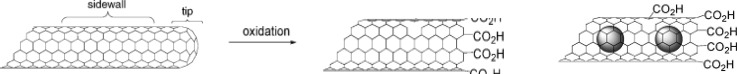
Structure showing the tips and sidewall zones of CNTs their reactivity

CNTs may exhibit extraordinary aspect ratios. SWNTs are found to grow up to several centimeters long, while MWNTs exhibit lengths of up to a centimeter and have diameters of 5-100 nm. From a chemical reactivity point of view, CNTs can be differentiated into tips and sidewall (curved part) zones (Figure 2). Tips are relatively reactive, while the reactivity of sidewalls is considerably lower than that of the tips. Therefore, more reactions are expected to occur at the tips first and then in some cases at the sidewalls. The difference in reactivity could result in a selective oxidation of the tips and consequently opening of the tubes with the tips consisting of oxygenated functions, mainly carboxylic acids. Another opportunity is the covalently attachment of molecular appendages to the CNT sidewalls. 

In principle, since the tips are open, a number of molecules could also be inserted in the internal space of CNT, mostly due to the hydrophobic interactions. The unique physical characteristics of CNTs are due to the strength of C-C bonds in the structure. Theoretically, CNTs are expected to be as hard as diamonds, but various investigations have indicated that they could be bent under a high pressure. In fact, the presence of sp^2^-hybridized carbon atoms and five-membered rings make these structures highly flexible. 

Results obtained by several researchers have shown that functionalization remarkably reduced the toxic effects of CNTs, while increasing their biocompatibility (the higher the degree of functionalization, the safer is the material), indicating the potential exploitation of nanotubes for drug administration. It has also been shown that functionalized, water soluble CNTs can be well tolerated *in-vivo *and taken up by cells to a considerable degree in an energy-independent manner and therefore, have a specific capacity to cross cell membranes. 

Carbon nanotubes are intrinsically poor soluble compounds, due to their rather hydrophobic character of the graphene sidewalls and strong interactions between the individual tubes. From the pharmaceutical point of view, it is essential that CNTs be dispersed before they are used in therapeutic formulations. Therefore, the solubility of CNTs in aqueous medium is a prerequisite for biocompatibility and CNT composites used in therapeutic delivery should meet this basic requirement. Beside the solubility obstacle, CNT dispersion in aqueous medium should also be stable and uniform. Four main approaches have been applied to overcome the dispersion problems, including use of solvent dispersion technique, functionalization of sidewalls, application of surfactant and use of biomolecular dispersion in complete dispersion. CNTs show no sign of aggregation and phase separation for several months, resulting in the formation of stable suspension. Among the above-mentioned approaches, covalent sidewall functionalization has been shown to produce the most stable dispersion, because the function of bound functional groups and the density of the bound groups significantly affect the dispersion. 

In general, what makes the CNTs quite unique is their ability to passively cross the membranes of many different types of cells. Additionally, it has been shown that adequately functionalized CNTs could be rapidly eliminated from the body following the systemic administration. The CNT safety profile could also be determined considering the excretion rates and accumulation in organs and any reactivity with the immune system. Although it seems too early to claim that CNTs are clinically successful therapeutics, these nanostructures are gradually playing a wider and more important role in the development of nanomedicines.

*Dr. Reza Aboofazeli is currently working as a Professor of Pharmaceutics at the Department of Pharmaceutics, School of Pharmacy, Shaheed Beheshti University of Medical Sciences, Tehran, Iran. He could be reached at the following e-mail address: raboofazeli@sbmu.ac.ir*

